# Secondary research use of personal medical data: patient attitudes towards data donation

**DOI:** 10.1186/s12910-021-00728-x

**Published:** 2021-12-15

**Authors:** Gesine Richter, Christoph Borzikowsky, Bimba Franziska Hoyer, Matthias Laudes, Michael Krawczak

**Affiliations:** 1 Institute of Experimental Medicine, Division of Biomedical Ethics, Kiel University, University Hospital Schleswig-Holstein, Niemannsweg 11, Haus 1, 24105 Kiel, Germany; 2 Institute of Medical Informatics und Statistics, Kiel University, University Hospital Schleswig-Holstein, Kiel, Germany; 3 Department of Internal Medicine 1, University Hospital Schleswig-Holstein, Kiel, Germany; 4 Division of Endocrinology, Diabetes and Clinical Nutrition, Department of Medicine 1, University Hospital Schleswig-Holstein, Kiel, Germany

**Keywords:** Data donation, Patient consent, Medical research, Secondary data use, Precision medicine, Public health

## Abstract

**Background:**

The SARS-CoV-2 pandemic has highlighted once more the great need for comprehensive access to, and uncomplicated use of, pre-existing patient data for medical research. Enabling secondary research-use of patient-data is a prerequisite for the efficient and sustainable promotion of translation and personalisation in medicine, and for the advancement of public-health. However, balancing the legitimate interests of scientists in broad and unrestricted data-access and the demand for individual autonomy, privacy and social justice is a great challenge for patient-based medical research.

**Methods:**

We therefore conducted two questionnaire-based surveys among North-German outpatients (n = 650) to determine their attitude towards data-donation for medical research, implemented as an opt-out-process.

**Results:**

We observed a high level of acceptance (75.0%), the most powerful predictor of a positive attitude towards data-donation was the conviction that every citizen has a duty to contribute to the improvement of medical research (> 80% of participants approving data-donation). Interestingly, patients distinguished sharply between research inside and outside the EU, despite a general awareness that universities and public research institutions cooperate with commercial companies, willingness to allow use of donated data by the latter was very low (7.1% to 29.1%, depending upon location of company). The most popular measures among interviewees to counteract reservations against commercial data-use were regulation by law (61.4%), stipulating in the process that data are not sold or resold (84.6%). A majority requested control of both the use (46.8%) and the protection (41.5%) of the data by independent bodies.

**Conclusions:**

In conclusion, data-donation for medical research, implemented as a combination of legal entitlement and easy-to-exercise-right to opt-out, was found to be widely supported by German patients and therefore warrants further consideration for a transposition into national law.

**Supplementary Information:**

The online version contains supplementary material available at 10.1186/s12910-021-00728-x.

## Background

Personal medical data are generated and stored in a variety of contexts, from clinical routine and medical research, via government agencies and insurance companies, to health apps and social media. The secondary use of such pre-existing data can greatly improve the quality, fairness and efficiency of both clinical care and medical research. However, the current legal-ethical framework of the processing of personal data is more prohibitive than supportive in this regard in many countries, including several EU member states.

Undoubtedly, the ever-increasing efficiency of digital technologies bears great potential for abridging the way from primary data generation to secondary data use, but it also brings with it the responsibility to adapt these processes to an increasing demand for patient autonomy and privacy as well as for social justice. On the other hand, the SARS-CoV-2 pandemic has highlighted that enabling efficient access, integration and use of patient data is an indispensable prerequisite, not only for promoting translation and personalisation as the two major goals of medical research, but also for fostering public health with regard to disease monitoring, prevention and the evaluation of political measures. Balancing these concerns represents a great challenge for patient-based data-driven medical research.

Various programmes have been implemented in different countries to enable access to, and ensure the interoperability of, personal medical data for research (e.g. MyHealthRecord in Australia, www.myhealthrecord.gov.au; FINDATA in Finland, www.findata.fi). In most of these instances, data provision is legitimized by the prior informed consent of the data subjects. However, patient consent is mostly obtained in clinical care situations, which is problematic for a number of reasons.

- Information and consent documents are usually handed out to patients during admission and alongside other documents relevant to their treatment. Moreover, for practicality reasons, information can realistically be given only in written form, with the help of other media, or orally by admission staff. However, even these options already entail a considerable demand of resources that is not easily met at large scale in routine care.
- During the consent process, patients are usually awaiting a serious diagnosis or therapy. In such situations, weighing the pros and cons of the secondary research use of their data places a significant extra burden upon patients. Moreover, there is a risk that the temporal and spatial association of the consent process with clinical care measures leads to therapeutic [1] or diagnostic misconception [2].
- It is inherently difficulty to create thorough understanding among patients of all aspects of the secondary research use of their medical data. At least in written form, this is rarely achievable within the time available, despite strong efforts to ensure readability and simple presentation [3, 4].

There is generally great willingness among patients to contribute to medical research by way of sharing personal data, mainly out of altruism, solidarity and an idea of reciprocity [5–8]. At the same time, however, it is not easy for individuals to enable and control data access by researchers directly, which limits patient autonomy [9]. In view of these imbalances, the German Ethics Council recently brought into play the concept of ‘data donation’ to allow individuals to better facilitate research use of their medical data [10, p. 266f]. Like others, the German Ethics Council understands data donation as consenting without temporal or factual restrictions provided that (a) the possible consequences of the data donation act are made sufficiently clear and (b) an appropriate infrastructure is in place to manage and protect the data.

First empirical studies have indicated that the great willingness among German patients to make personal data available for medical research may even extend to a model without explicit consent, if necessary. In fact, a representative survey of the German population revealed an approval rate of almost 80% to the donation of own medical data for medical research without attaching the donation act explicitly to prior consent [11].

The way in which decision-making for or against data donation is implemented (i.e. opt-in or opt-out) has major impact upon the scientific value of the data in question. With an opt-in model, participation rates can be expected to be rather low, leading to bias that may not only render the data useless for research but that may ultimately result in erroneous and, hence, potentially dangerous scientific conclusions. Moreover, some important types of patients could get lost to medical research in this scenario, including emergency cases as well as patients who are incapable of giving consent in the first place. Therefore, the German Federal Ministry of Health commissioned an expert opinion in 2019 on the current legal basis for data donation in Germany, designed so that it would exempt secondary data use for medical research from the requirement of informed prior consent and instead grants citizens an easy-to-exercise right to opt-out [12]. In their report to the Ministry, the authors concluded that such a framework would not only be compliant with the provisions of the EU General Data Protection Regulation (2016/679) but could also be made ethically acceptable through suitable outreach and trust-building measures.

To our knowledge, our study is the first in Germany to evaluate patient views of a mechanism of data donation that stipulates an opt-out scenario. In addition to assessing the level of acceptance of such a model, we also sought to explore further the negative attitude of patients towards the research use of their data by commercial companies, as identified in earlier studies [11, 13], and how reservations against such use could be counteracted when implementing data donation in practice.

## Materials and methods

### Study participants

Our study was conducted in the form of two questionnaire-based surveys, one to identify predictors of a positive attitude towards data donation (Survey 1), and one to verify and specify further these predictors (Survey 2) using a questionnaire built upon the results of Survey 1. The full texts of both questionnaires (in German) are provided in the Additional file 1.

After approval by the local ethics committee, the two surveys were undertaken between May and November 2020, including 500 and 150 patients, respectively. Participants were approached in the waiting rooms of the Comprehensive Center for Inflammation Medicine (CCIM) and the joint Outpatient Center of the Departments of Internal Medicine and General Surgery (IMAC) at University Hospital Schleswig–Holstein (UKSH) Campus Kiel. The CCIM and IMAC patient populations adequately reflect the socio-demographic structure of all outpatients at UKSH Campus Kiel, and of the general population of the most northern part of Germany.

### Survey 1

The questionnaire used in Survey 1 focussed upon the personal attitude of participants towards medical research (Section A) and data donation (Section B). Section A comprised 12 statement items addressing the understanding and personal views of medical research in general, including the potential duty to contribute to medical research and the role of commercial companies, among others. Answering options were formulated on either a three-graded Likert scale (1: “yes”, 2: “no”, 3: “unknown”) or a four-graded Likert scale (1: “fully agree”, 2: “rather agree”, 3: “rather disagree”, 4: “fully disagree”). Section B comprised (i) two four-graded Likert scale questions about the acceptance of data donation, (ii) five statement items concerning the possible implementation of data donation (site of data storage, rights-of-use, reservations against commercial data use, countermeasures against such reservations, context of consent decision-making), and (iii) two questions about whether the SARS-CoV-2 pandemic affected the participant’s attitude towards data donation and the research use of their data by commercial companies.

### Survey 2

The questionnaire of Survey 2 was intended (i) to verify major results of Survey 1 and (ii) to address more specifically aspects of a potential practical implementation of data donation. It comprised two four-graded Likert scale questions about (i) the participant’s attitude towards legally permitting research use of pseudonymised medical data without prior consent (i.e. an opt-out implementation of data donation) and (ii) their agreement, or not, to a general civic duty to improve medical research (i.e. the main predictor of a positive attitude towards data donation identified in Survey 1). In addition, the two statement items in Survey 1 regarding storage site and rights-of-use were also included in Survey 2. However, the rights-of-use item was slightly refined so as to differentiate between use within and outside the EU (rather than within and outside Germany, as in Survey 1).

### Statistics

All statistical analyses were carried out with IBM SPSS Statistics for Windows [14]. The variables considered in our study were of categorical type (e.g., age class, agreement to a given statement) and were characterized by their absolute and relative class frequencies. Chi-squared (χ^2^) or Fisher’s exact tests were used, as appropriate, to assess the statistically significance of frequency differences between groups of participants (e.g., proponents and opponents of data donation). Predictors of the approval, or otherwise, of data donation were identified by step-wise logistic regression analysis with forward selection, using a Wald test to ascertain non-zero regression coefficients. *p* values smaller than 0.05 were regarded as statistically significant.

## Results

Of the 500 questionnaires delivered in Survey 1, 376 (75.2%) were returned at a level of completeness sufficient for subsequent analysis. Of the 150 questionnaires delivered in Survey 2, 132 (88.0%) were returned in appropriate form. By and large, the two samples were representative of the general German population in terms of age and sex (Table 1), with one exception: The proportion of participants who did not rise above primary or secondary school level was significantly higher in Survey 2 (37.1%) than in Survey 1 (21.0%; χ^2^ = 12.613, 1 df, p = 3.8 × 10^–4^), with both percentages deviating by roughly the same amount from the 2019 nation-wide figure of 28.6% [15].

**Table 1:** Attitude towards data donation according to age, sex and education (school leaving certificate)

Age (years), Education	Male (%)	Pro data-donation (%)^a^	Female (%)	Pro data-donation (%)^a^
*Survey 1*				
18–25	13 (8.4)	13 (100.0)	16 (7.2)	9 (56.3)
26–30	11 (7.1)	6 (54.5)	18 (8.1)	13 (72.2)
31–40	30 (19.5)	24 (80.0)	44 (19.8)	32 (72.7)
41–50	30 (19.5)	24 (80.0)	42 (18.9)	30 (71.4)
51–65	44 (28.6)	38 (86.4)	70 (31.5)	59 (84.3)
66–75	17 (11.0)	16 (94.1)	22 (9.9)	15 (68.2)
Over 75	9 (5.8)	9 (100.0)	10 (4.5)	8 (80.0)
Primary/ secondary school	35 (22.7)	27 (77.1)	44 (19.8)	34 (77.3)
Middle school	66 (42.9)	62 (93.9)	97 (43.7)	74 (76.3)
High school	34 (22.1)	26 (76.5)	60 (27.0)	40 (66.7)
Other	16 (10.4)	12 (75.0)	14 (6.3)	11 (78.6)
None	3 (1.9)	3 (100.0)	5 (2.3)	5 (100.0)
No answer	0	0	2 (0.9)	2 (100.0)
Total (%)^b^	154 (41.0)	130 (43.9)	222 (59.0)	166 (56.1)
*Survey 2*				
18–25	4 (7.5)	4 (100.0)	2 (2.5)	2 (100.0)
26–30	6 (11.3)	5 (83.3)	6 (7.6)	4 (66.7)
31–40	6 (11.3)	3 (50.0)	16 (20.3)	12 (75.0)
41–50	9 (17.0)	6 (66.7)	21 (26.6)	13 (61.9)
51–65	19 (35.8)	15 (78.9)	27 (34.2)	22 (81.5)
66–75	6 (11.3)	5 (83.3)	4 (5.1)	2 (50.0)
Over 75	3 (5.7)	3 (100.0)	2 (2.5)	2 (100.0)
No answer	0	0	1 (1.3)	1 (100.0)
Primary/secondary school	25 (47.2)	18 (72.0)	24 (30.4)	18 (75.0)
Middle school	13 (24.5)	10 (76.9)	34 (43.0)	28 (82.4)
High school	14 (26.4)	12 (85.7)	16 (20.3)	9 (56.3)
Other	1 (1.9)	1 (100.0)	2 (2.5)	2 (100.0)
None	0	0	3 (3.8)	1 (33.3)
Total (%)^b^	53 (40.2)	41 (77.4)	79 (59.8)	58 (73.4)

^a^Percentage of participants in sex-specific age or education group who answered “Do fully agree” or “Do rather agree” to Q5 (Survey 1) or Q3 (Survey 2)

^b^Percentage of all participants

### Acceptance of data donation

**Fig. 1:**
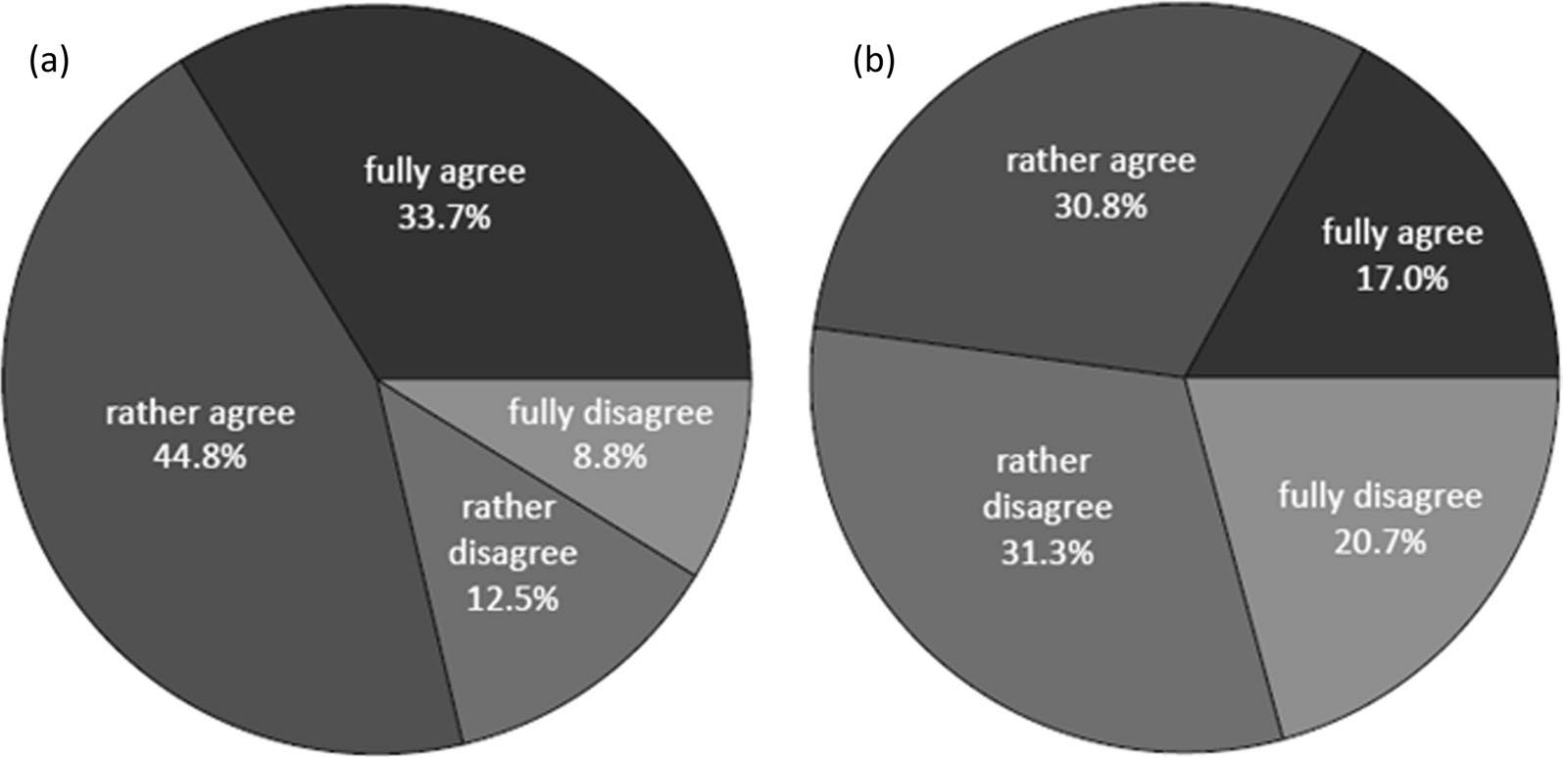
Attitude of participants in Survey 1 (n = 376) towards donation for medical research of **a** data from EHRs (S1/Q5) and **b** self-acquired medical data (S1/Q6). S1/Q5: “In the future, your personal health data will likely be stored in a digital health record. Would you agree that these data become available for medical research as a ‘data donation’, free of charge and in compliance with data protection laws, without asking for your permission prior to each use of the data?” S1/Q6: “Would you agree that data collected by yourself (e.g. via medical devices or mobile phones) are made available to medical research as a ‘data donation’, free of charge and in compliance with data protection regulations, without asking for your permission prior to each use of the data?”

In Survey 1, the overall attitude towards data donation for medical research was ascertained by asking participants whether they would approve use of their data, free of charge and in compliance with pending data protection regulations, but without having to ask them for permission each time the data were used (question S1/Q5, Additional file 1: Table S1). With this definition, the donation of medical data from own electronic health records (EHRs, e.g. examination results, anamneses, X-rays) was deemed acceptable by 296 participants (78.7%), who answered “Do fully agree” or “Do rather agree” to question S1/Q5 (henceforth referred to a s the ‘pro’ data donation subgroup), and was opposed by 80 participants (21.3%), who answered “Do rather disagree” or “Do fully disagree” (‘contra’ data donation; Fig. 1a). With regard to the donation of self-acquired data, such as those generated by medical devices or mobile phones (S1/Q6), positive (n = 180, 47.8%) and negative attitudes (n = 196, 52.0%) were found to be balanced among participants (Fig. 1b). Interestingly, a vast majority of participants reported that the SARS-CoV-2 pandemic had had no effect upon their attitude towards data donation (n = 289, 76.9%; S1/Q12).

**Fig. 2:**
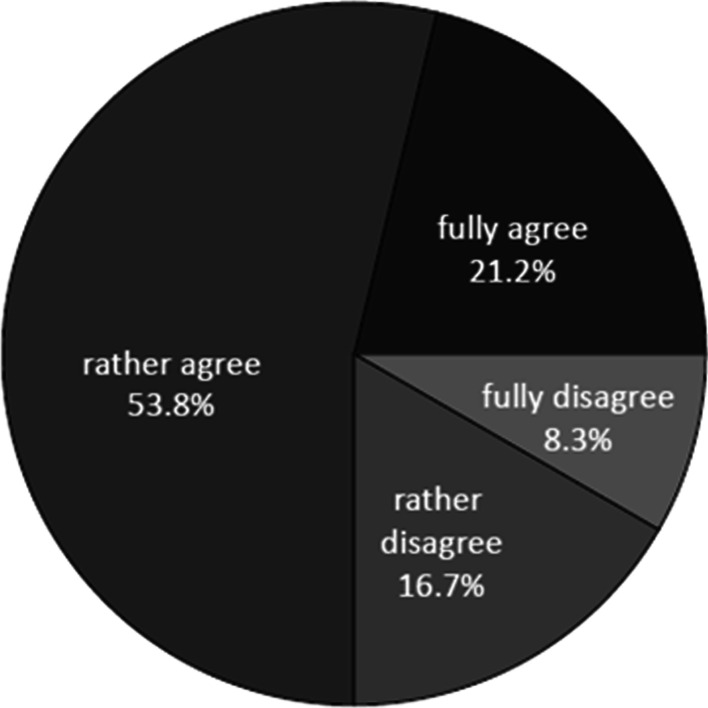
Attitude of participants in Survey 2 (n = 132) towards donation for medical research, implemented as an opt-out process (S2/Q3). S2/Q3: “There are currently considerations in Germany to legally allow medical research on pseudonymised data without the prior consent of patients, unless the patient objects to such use. The objection should be as simple as possible, e.g. possible to assert when visiting a doctor or a pharmacy. Would you agree to such a regulation?”

For Survey 2, the question about the general attitude towards data donation (S2/Q3) was modified so as to address the strong demand expressed in Survey 1 for a legal regulation of the donation process (see below). The revised wording therefore implied that data donation comprised a legal entitlement of researchers to use patient data without prior consent, but that the entitlement was combined with a simple opt-out for patients. Even under these more liberal conditions, the rate of acceptance of data donation (n = 99, 75.0%; Fig. 2, Additional file 1: Table S2) was almost as high as in Survey 1. In both surveys, no correlation was observed between gender, age or educational level and the participants’ attitude towards data donation (Table 1).

### General understanding of medical research (Survey 1)

In Survey 1, participants were first asked about their general understanding of medical research (Additional file 1: Table S1, S1/Q3). A vast majority agreed that data from routine clinical care can be useful for research (n = 322, 85.6%). When asked whether a legal entitlement, if any, to the research use of such data without prior consent of the patient was limited to the treating physicians, a majority rightly thought that this was the case (n = 209, 55.6%). Interestingly, such a ‘privilege of own research’ is a reality in almost all German federal states (‘Länder’), albeit not in Schleswig–Holstein where our study was carried out. A majority still was rightly aware that universities cooperate with commercial companies in medical research (n = 222, 59.0%), and that conclusions about individual patients cannot easily be drawn from scientific publications (n = 226, 60.1%). However, more than 20% of the participants found themselves unable to answer the latter two questions in the first place, with a somewhat higher level of indecisiveness noted in the ‘contra’ subgroup (32.5%, 31.3%) than in the ‘pro’ subgroup (29.1%, 20.3%). Even greater uncertainty prevailed about whether medical data that are used in research can be traced back to patients. Here, all three answering options (“yes”, “no”, “unknown”) were chosen with roughly equal frequency. Notably, a significantly higher proportion of participants in the ‘pro’ subgroup (n = 135, 45.6%) than in the ‘contra’ subgroup (n = 19, 23.8%; χ^2^ = 11.555, 1 df, p = 6.8 × 10^–4^) was aware of the fact that data from German patients may also be used for research abroad.

### Personal attitude towards medical research

Most participants in both the ‘pro’ and ‘contra’ subgroup of Survey 1 took the so-called ‘reciprocity’ position that patients who benefit from medical research should contribute to research themselves (Additional file 1: Table S1, S1/Q4; pro: n = 267, 90.2%; contra: n = 64, 80.0%; χ^2^ = 5.292, 1 df, p = 0.0214). A highly significant difference became apparent, however, when participants were asked for whether every citizen (diseased, or not) has a duty to contribute to the improvement of medical research. While a vast majority of those supporting data donation shared this view (n = 247, 83.4%), just over half the opponents either “fully” or “rather” agreed to the respective statement (n = 45, 56.3%; χ^2^ = 25.307, 1 df, p < 10^–5^). While both subgroups were found in Survey 1 to be quite indifferent towards the possible entitlement to personal benefits of patients involved in medical research (pro: n = 155, 52.4%; contra: n = 33, 41.3%, non-significant), supporters of data donation were significantly more positive about the availability of patient data for medical research to commercial companies (n = 167, 54.4%) than those opposing data donation (n = 20, 25.0%; χ^2^ = 23.628, 1 df, p < 10^–5^). Notably, a majority in both subgroups rejected the idea that outsiders such as self-help groups, churches or charities should be involved in deciding about the research use of patient data, with a significantly less negative attitude in this regard prevailing in the ‘pro’ subgroup (n = 161, 54.4%) than in the ‘contra’ subgroup (n = 59, 73.8%; χ^2^ = 8.941, 1 df, p = 0.0028). Finally, a vast majority of both subgroups shared a demand for more public information on individual research projects (pro: n = 274, 92.6%; contra: n = 73, 91.3%, non-significant).

That the conviction of a civic duty to advance medical research is a strong predictor of the attitude towards data donation was confirmed in Survey 2 (Additional file 1: Table S2, S2/Q4). There, a vast majority of 90 participants in the ‘pro’ subgroup (90.9%) agreed to the supposition of such a duty, compared to only 13 participants in the contra subgroup (39.4%; χ^2^ = 35.368, 1 df, p < 10^–5^).

### Framework of data donation

#### Site of data storage

In both surveys, most participants from the ‘pro’ subgroup favoured storage of their donated data in a nation-wide centralized database (S1/Q7: n = 214, 72.3%, Additional file 1: Table S1; S2/Q5: n = 75, 75.8%, Additional file 1: Table S2). The second most favourite option was storage exclusively at the site of original data acquisition (Survey 1: n = 131, 44.3%; Survey 2: n = 37, 37.4%). Long-term storage at the research institutions that use the data was found to be acceptable only to a minority of participants (Survey 1: n = 110, 37.2%, Survey 2: n = 23, 23.2%).

#### Rights-of-use

**Fig. 3:**
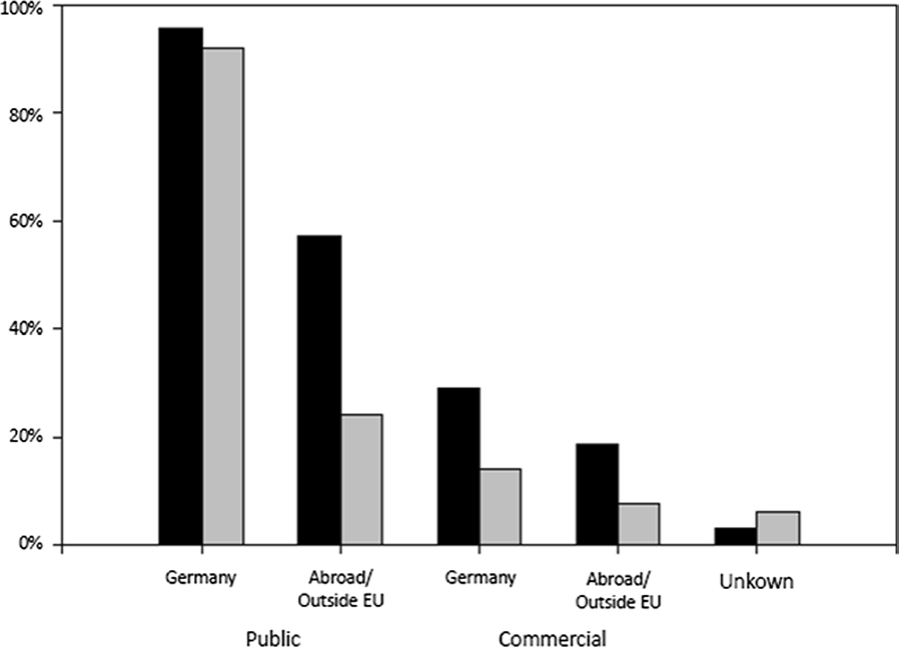
Rights-of-use granted by proponents of data donation in Survey 1 (S1/Q8; n = 296; black bars) and Survey 2 (S2/Q6; n = 99; grey bars). S1/Q8, S2/Q6: “Who should be allowed use of data donated for medical research (multiple answers possible)?”

In both surveys, participants were asked who should be allowed research use of their donated data (Fig. 3). In Survey 1, the respective question (S1/Q8) aimed at differentiating between public and commercial research institutions as well as between institutions within and outside Germany. In order to account more adequately for the legal framework of data donation in the EU and the current practise of data sharing in medical research in general, however, we slightly modified this question in Survey 2 so as to distinguish between institutions inside and outside the EU (S2/Q6). The vast majority of patients in the ‘pro’ subgroup was willing to allow data use by universities and public research institutions in Germany (Survey 1: n = 284, 95.9%) and in the EU (Survey 2, n = 91, 91.9%). When data usage abroad was specified as meaning ‘outside the EU’ in Survey 2, only a minority was still approving data access by universities and public research institutions there (n = 24, 24.2%). Drastically fewer participants were willing to accept data access by commercial companies, irrespective of whether they were located in Germany (Survey 1: n = 86, 29.1%), inside the EU (Survey 2: n = 14, 14.1%), or outside the EU (Survey 2: n = 7, 7.1%). All reductions in approval, compared to universities and public research institutions, were statistically highly significant (p < 10^–5^).

#### Reservations against commercial data usage, and appropriate countermeasures

Previous research by ourselves [11] had unveiled reservations against the research use of personal medical data by commercial institutions. In Survey 1, we therefore asked patients who felt that they had such reservations in the first place, or who opposed data donation in general, for the precise nature of their reservations (n = 219, S1/Q9). The most frequently chosen items were fear of insufficient data protection by commercial companies (n = 150, 68.5%) and rejection of profit-making through the research use of patient data (n = 123, 56.2%). Doubt whether commercial companies would do research for the common good was less important (n = 86, 39.3%). This notwithstanding, there was an overwhelming acknowledgment among participants of the importance of commercial medical research, which was in fact questioned by only a small minority (n = 14, 6.4%).

Among the participants of Survey 1, the most popular measures to counteract potential reservations against the research use of patient data by commercial companies (S1/Q10) were an assurance that the data are not sold or resold (n = 318, 84.6%) and a legal regulation of the conditions for using the data (n = 231, 61.4%). A slightly smaller proportion of participants would have wished for research usage of the data to be controlled by an independent body (n = 176, 46.8%) and for the data protection measures taken by commercial companies to be monitored regularly and independently (n = 156, 41.5%). Other counter measures such as controlling data usage by state institutions (n = 127, 33.8%), the public availability of information on the data usage (n = 91, 24.2%), or a ban on data storage within the companies themselves (n = 88, 23.4%) were of minor importance.

#### Context of decision-making

The circumstances under which a patient is asked for their consent to the research use of their medical data is likely to impact upon both, the understanding of the information provided and the actual decision-making process. When participants of Survey 1 were asked about their preferences in this regard (S1/Q11), however, a majority answered that they would rather want to undergo the consent process as a patient in the clinic (n = 251, 66.8%). Only a minority wanted to make this decision outside the clinic (n = 50, 13.3%) or before they became ill at all (n = 56, 14.9%).

### Predictors of attitude towards data donation

In Survey 1, logistic regression analysis with forward selection was carried out to formally identify predictors of a positive patient attitude towards data donation among the six possible responses to question S1/Q4 (“What is your personal attitude towards medical research?”; Additional file 1: Table S1). The logistic regression analysis was adjusted for sex, age and highest level of education, but none of these covariates had a statistically significant influence upon the approval, or not, of data donation from EHRs (S1/Q5). In contrast, both an agreement to the civic duty to contribute to medical research (S1/Q4, item 2) and an approval of the research use of patient data by commercial companies (item 6) were significantly associated with a positive attitude (Table 2). The same predictors emerged for an approval of the donation of self-acquired medical data (S1/Q6), in addition to male sex which was interestingly found to be a positive predictor with an odds ratio of almost two (Table 2). Survey 2 was undertaken to determine more specifically the acceptance by patients of an opt-out implementation of data donation (S2/Q3), and the civic duty to support research (S2/Q4) was as an even stronger positive predictor here than in Survey 1 (Table 2). In contrast to Survey 1, however, agreement to the research use of donated data by commercial companies (implied by a positive response to item 3 or item 4, or both, of question S2/Q6) was not significantly associated with an approval of data donation.

**Table 2:** Logistic regression analysis of patient attitude towards data donation

Outcome, Predictor	OR (95% CI)	W	P
*Data donation from electronic health records (S1/Q5)*			
S1/Q4-2 (civic duty to contribute to medical research)	3.206 [1.803; 5.699]	15.747	< 10^–5^
S1/Q4-6 (research use of patient data by commercial companies)	2.908 [1.625; 5.203]	12.921	< 10^–5^
*Donation of self-acquired medical data (S1/Q6)*			
Sex (male)	1.876 [1.194; 2.945]	7.460	0.006
S1/Q4-2 (civic duty to contribute to medical research)	2.035 [1.129; 3.665]	5.594	0.018
S1/Q4-6 (research use of patient data by commercial companies)	3.161 [2.008; 4.976]	24.710	< 10^–5^
*Opt-out implementation of data donation (S2/Q3)*			
S2/Q4 (civic duty to contribute to medical research)	15.214 [5.718; 40.478]	29.728	< 10^–5^

OR, odds ratio (“fully agree” or “rather agree” *versus* “rather disagree” or “fully disagree”); CI, confidence interval; W, Wald test statistic (OR = 1); P, *p* value (from a χ^2^ distribution with 1 df)

## Limitations

### Influence of the SARS-CoV-2 pandemic

The present study was undertaken between May and November 2020. Particularly during Survey 1, there was thus considerable uncertainty among patients about the pandemic-related conditions of their hospital stay. At the same time, medical research got more into the focus of public interest. Patients understood better that researchers in public and commercial institutions need to cooperate, both nationally and internationally, and the benefits of such cooperation for the public good became tangible by the rapid development of vaccines. Against this background, it cannot be excluded that the level of approval of data donation expressed in both surveys was influenced by the acute pandemic situation. However, when explicitly asked in Survey 1 for the possibility of a change of mind due to the pandemic (S1/Q11-Q12; Additional file 1: Table S1), a majority of patients in both the ‘pro’ and the ‘contra’ subgroup claimed that the pandemic had had no significant influence upon their attitudes, neither towards data donation (n = 289, 76.9%) nor towards the research use of medical data by commercial research (n = 301, 80.1%). This notwithstanding, an unconscious influence cannot be completely ruled out.

### Survey setting

Our study involved patients waiting for counselling or treatment in a UKSH outpatient unit. Therefore, it is possible that the answers given to question S1/Q11 about the preferred context of decision-making were biased by the setting of the survey itself. Indeed, to our surprise, a great majority in both subgroups (‘pro’ and ‘contra’) was found to be in favour of being asked for consent to the research use of their medical data in the clinic, and as a patient.

## Discussion

The SARS-CoV-2 pandemic has highlighted once more the great need for comprehensive access to, and uncomplicated use of, pre-existing patient data for medical research. Enabling the secondary use of such data is an indispensable prerequisite for the promotion of translation and personalisation in medicine and for the advancement of public health, none of which can be achieved by prospective studies alone. However, balancing the legitimate interests of scientists as well as current and future patients in broad and unrestricted data access for research on the one hand, and the demand for individual autonomy, privacy and social justice on the other, is a great challenge for patient-based medical research. Successfully meeting this task requires a broad understanding of benefits and risks that is both personal and societal [16, p.122], a view that renders ‘data donation’, implemented as a legal entitlement to both, the use of data and the objection to such use, a potentially widely acceptable option in medical research.

Recent surveys undertaken against the backdrop of the SARS-CoV-2 pandemic revealed that a large majority of German citizens would be prepared to make personal health data available for research under the prevailing exceptional circumstances, including research by private companies [17, 18]. Even before the pandemic, studies in various countries [19–21] demonstrated high willingness of patients and members of the general population to contribute personal data to medical research more generally. Not surprisingly then, initial considerations have gotten under way since then to create a legal basis in Germany for data donation in the manner described [12, 22, 23]. In fact, previous studies of ours [8, 11] have suggested robust acceptance at the national level of data donation if understood as one of many possible forms of patient consent, but the envisaged legal and organizational concept was not differentiated in these surveys in much detail.

Our present study therefore aimed to determine the attitude of patients towards the specific design of data donation for medical research as an opt-out process. As a result, we observed a high level of acceptance, and by far the most powerful predictor of a positive attitude towards such a model was the conviction that every citizen (whether diseased or not) has a duty to contribute to the improvement of medical research. Notably, a recent study in the UK made very similar observations albeit for data donation as an opt-in process [24]. Nevertheless, the considerable agreement between the two studies suggests that the assumption, in Survey 2 of our study, of a legal stipulation did not particularly foster the view of participants who backed data donation that participation in the process were a civic duty.

Most patients included in our study were well aware that data from routine clinical care can be useful for medical research. Their high willingness to contribute such data to research was likely motivated by a so-called ‘reciprocity’ position which implies that patients who benefit from medical research should contribute to research themselves, an attitude encountered in many studies before [25]. The most prominent (potential) beneficiaries of such patient generosity were universities and public research institutions in Germany. Despite a general awareness that universities and public research institutions cooperate with commercial companies, willingness to allow use of the donated data by the latter was very low.

In view of this apparent contradiction, which agrees with results by other studies [26–30], we tried to identify the actual reasons for the observed reservations and to find out how they could be counteracted when designing a data donation process. According to Survey 1, the most frequent concerns are insufficient data protection by commercial companies and an objection of their profit-making through the use of the data. The view that commercial companies would not conduct research for the common good, as implied occasionally in the literature [28, 31], played only a minor role in our study. Consequently, the most popular measure to counteract reservations on the side of patients was regulation of the commercial use of the data by law, stipulating in the process that the data are not sold or resold. Additionally, patients requested control of both the use and the protection of the data by independent bodies.

In their attitude towards data donation, patients distinguished sharply between research use of their data inside and outside the EU, particularly when commercial companies were involved. Since people in Germany have one of the highest levels in Europe of concern about data protection in general [32], this distinction most likely reflects greater trust in data protection measures taken inside than outside Europe, combined with the wide-spread mistrust in data protection by commercial institutions as alluded to above. This unwillingness may have been reinforced further by an inadequate perception of legal and administrative hurdles. In fact, the pros and cons of transferring personal data to outside the EU are likely unknown to most parts of the general population and, as was pointed out earlier, it is difficult to put an end to this ignorance under the conditions of current patient consent practice. Moreover, most people are likely unaware that commercial companies have good reason to work with patient data and are more than willing to benefit the original causes of data donation, for example, by transferring back their research results for use by others. In any case, if data donation is to be a success, it is the joint responsibility of politics and science to explain these aspects of sharing donated data better to citizens in order to reduce their potential fear of it.

A main motivation for contemplating, in Germany, data donation for medical research as a combination of legal entitlement and opt-out was the prospect of being able to separate the decision-making process of patients from a clinical care context [12]. This way, problems in comprehending the consent documents as well as possible therapeutic or diagnostic misconceptions could be avoided ‘by design’ through relaying the necessary information about data donation before the individuals concerned become ill and, hence, biased in terms of their personal perception and attitude. When preparing our study, we expected this prospective increase in fairness, associated with decoupling consent and medical treatment, to greatly appeal to participants. In contrast, however, a majority was found to wish to continue deciding about the research use of their data as patients in the clinic. Since it cannot be excluded that this observation reflects bias due to the survey context itself (see Limitations), additional studies outside the clinic seem well warranted in the future.

## Conclusion

Our study strongly suggests that data donation for medical research, implemented as a combination of legal entitlement and an easy-to-exercise right to opt-out, is widely supported by German patients and therefore warrants further consideration for a transposition into national law. We observed that the most powerful predictor of a positive attitude towards data-donation is the conviction that every citizen is obliged to contribute to the improvement of medical research. Hence, endorsing and supporting such a sense of civic duty by way of better public outreach and stakeholder involvement may be a key for medical research to sustain the secondary use of patient data. Despite a general awareness that academic institutions cooperate with commercial companies, the willingness of participants in our study to allow use of their donated data by industry was found to be low. These reservations should be understood as a mandate to rethink the legal framework of the processing of personal medical data in research because it appears as if the reservation can be successfully counteracted by a strong legal ban on selling or reselling the data. In conclusion, we trust that our study will stimulate further the debate about the most efficient and, at the same time, ethically acceptable use of patient data for research purposes.

## Supplementary Information

**Additional file 1:** **Questionnaire 1.** Questionnaire of Survey 1. **Questionnaire 2.** Questionnaire of Survey 2.

## Data Availability

Data supporting the results reported in the article can be found in the supplements added.
