# Supplementary material for: Secondary research use of personal medical data: patient attitudes towards data donation
Source: BMC Med Ethics. 2021 Dec 15;22:164. doi: 10.1186/s12910-021-00728-x (PMC8672332; doi:10.1186/s12910-021-00728-x)
Supplement: Supplementary file 1 — Additional file 1: Questionnaire 1. Questionnaire of Survey 1. Questionnaire 2. Questionnaire of Survey 2. [file 12910_2021_728_MOESM1_ESM.docx]

Supplementary Table 1: Results of Survey 1

| **Survey 1 Q3: In your opinion, which of the following statements about medical research do currently apply?** | Pro data donation^a^ (n=296) | | | Contra data donation^a^ (n=80) | | |
| --- | --- | --- | --- | --- | --- | --- |
|  | Yes | No | Unknown | Yes | No | Unknown |
| Data from routine clinical care are useful for medical research. | 261 (88.2%) | 4 (1.4%) | 31 (10.5%) | 61 (76.3%) | 4 (5.0%) | 15 (18.8%) |
| Without explicit consent, patient data may only be used for research by the treating physicians. | 171 (57.8%) | 101 (34.1%) | 24 (8.1%) | 38 (47.5%) | 33 (41.3%) | 9 (11.3%) |
| Data from German patients can also be used for research abroad. | 135 (45.6%) | 110 (34.2%) | 51 (17.2%) | 19 (23.8%) | 40 (50.0%) | 21 (26.3%) |
| Universities cooperate with commercial companies in medical research. | 178 (60.1%) | 32 (10.8%) | 86 (29.1%) | 44 (55.0%) | 10 (12.5%) | 26 (32.5%) |
| Scientific publications allow conclusions to be drawn about individual patients. | 58 (19.6%) | 178 (60.1%) | 60 (20.3%) | 7 (8.8%) | 48 (60.0%) | 25 (31.3%) |
| Medical research data cannot be traced back to individual patients.^b^ | 106 (35.8%) | 74 (25.0%) | 115 (38.9%) | 28 (35.0%) | 21 (26.3%) | 31 (38.8%) |

^a^ For the purpose of the analysis of the survey 1 results, the 296 patients who answered either “Do fully agree” or “Do rather agree” to Q5 were categorized as ‘pro data donation’ whereas the 80 patients who answered “Do rather disagree” or “Do fully disagree” were categorized as ‘contra data donation’.

^b^ One patient in the ‘pro data donation’ subgroup did not answer this question.

| **Survey 1 Q4: What is your personal attitude towards medical research?** | Pro data donation (n=296) | | | Contra data donation (n=80) | | |
| --- | --- | --- | --- | --- | --- | --- |
|  | Fully/rather agree | Fully/rather disagree | No answer | Fully/rather agree | Fully/rather disagree | No answer |
| Patients who benefit from medical research should contribute to such research themselves. | 267 (90.2%) | 29 (9.8%) | -.- | 64 (80.0%) | 16 (20.0%) | -.- |
| Every citizen (diseased, or not) has a duty to contribute to medical research. | 247 (83.5%) | 45 (15.1%) | 4 (1.4%) | 45 (56.3%) | 35 (43.8%) | -.- |
| Medical research should personally benefit the patients involved. | 155 (52.4%) | 140 (47.3%) | 1 (0.3%) | 33 (41.3%) | 47 (58.75%) | -.- |
| More information on individual research projects should be made available to the public. | 274 (92.6%) | 21 (7.1%) | 1 (0.3%) | 73 (91.3%) | 7 (8.75%) | -.- |
| The use of patient data for medical research must not be decided by scientists alone, but should also involve externals (e.g. self-aid groups, churches or charities). | 135 (45.6%) | 161 (54.4%) | -.- | 21 (26.3%) | 59 (73.8%) | -.- |
| Patient data should also be made available for medical research by commercial companies. | 167 (54.4%) | 129 (43.6%) | -.- | 20 (25.0%) | 59 (73.8%) | 1 (1.3%) |

| **Survey 1 Q5:** **In the future, your personal health data will likely be stored in a digital health record. Would you agree that these data become available for medical research as a ‘data donation’, free of charge and in compliance with data protection laws, without asking for your permission prior to each use of the data?** | Fully agree | Rather agree | Rather disagree | Fully disagree |
| --- | --- | --- | --- | --- |
|  | 127 (33.7%) | 169 (44.8%) | 47 (12.5%) | 33 (8.8%) |

| **Survey 1 Q6:** **Would you agree that data collected by yourself (e.g. via medical devices or mobile phones) are made available to medical research as a ‘data donation’, free of charge and in compliance with data protection regulations, without asking for your permission prior to each use of the data?** | Fully agree | Rather agree | Rather disagree | Fully disagree |
| --- | --- | --- | --- | --- |
|  | 64 (17.0%) | 116 (30.8%) | 118 (31.3%) | 78 (20.7%) |

| **Survey 1 Q7: Where should the medical data that you donated for medical research be stored (multiple answers possible)?^a^** | Site of original data acquisition only (e.g. clinic, GP) | Nation-wide centralized database | Respective research institution | Nowhere (owing to rejection of data donation) | Unknown |
| --- | --- | --- | --- | --- | --- |
|  | 131 (44.3%) | 214 (72.3%) | 110 (37.2%) | 3 (1.0%) | 11 (3.7%) |

^a^ Only the responses of the 296 patients in the ‘pro data donation’ subgroup are shown.

| **Survey 1 Q8****: Who should be allowed use of data donated for medical research (multiple answers possible)?^a^** | Universities and public research institutions | | Commercial research institutions (pharma companies, insurance companies) | | Nobody (owing to rejection of data donation) | Unknown |
| --- | --- | --- | --- | --- | --- | --- |
|  | Germany | Abroad | Germany | Abroad |  |  |
|  | 284 (95.9%) | 169 (57.1%) | 86 (29.1%) | 55 (18.6%) | 1 (0.3%) | 9 (3.0%) |

^a^ Only the responses of the 296 patients in the ‘pro data donation’ subgroup are shown.

| **Survey 1 Q9: What reservations, if any, do you have against the research use of your medical data by commercial institutions (multiple answers possible)?^a^** | Insufficient data protection | Profit-making through data usage | No research for common good | Insufficient importance of research | Rejection of data donation in principle |
| --- | --- | --- | --- | --- | --- |
|  | 150 (68.5%) | 123 (56.2%) | 86 (39.3%) | 14 (6.4%) | 6 (2.7%) |

^a^ Percentages relate to those 219 patients only who expressed that they had any reservations at all.

| **Survey 1 Q10: How could possible reservations against the research use of donated medical data by commercial institutions be counteracted (multiple answers possible)?** | Pro data donation (n=296) | Contra data donation (n=80) |
| --- | --- | --- |
| It must be ensured that the data are not sold or resold. | 249 (84.1%) | 69 (86.3%) |
| The conditions for using the data must be regulated by law. | 189 (63.9%) | 42 (52.5%) |
| The use of data must be controlled by an independent body. | 140 (47.3%) | 36 (45.0%) |
| The use of the data must be controlled by a state institution. | 103 (34.8%) | 24 (30.0%) |
| Information on data use must be publicly available (e.g. in a register). | 75 (25.3%) | 16 (20.0%) |
| Data protection by such companies must be independently monitored regularly. | 123 (41.6%) | 33 (41.3%) |
| The data must not be stored in the companies themselves. | 55 (18.6%) | 33 (41.3%) |

| **Survey 1 Q11: So far, patients have been asked for consent to the research use of their medical data in the clinic, prior to treatment. Do you find this acceptable or would you rather wish to make this decision on a different occasion?** | Pro data donation (n=296) | Contra data donation (n=80) |
| --- | --- | --- |
| I would want to continue making this decision as a patient in the clinic. | 190 (64.2%) | 61 (76.3%) |
| I would want to make this decision when I am a patient, but outside the clinic. | 46 (15.5%) | 4 (5.0%) |
| I want to make this decision before I become a patient | 44 (14.9%) | 12 (15.0%) |
| No answer | 16 (5.4%) | 3 (3.8%) |

| **Survey 1 Q12-Q13: How has the corona pandemic changed your attitude towards …** | Pro data donation (n=296)^a^ | | | Contra data donation (n=80) | | |
| --- | --- | --- | --- | --- | --- | --- |
|  | Positive | Negative | No change | Positive | Negative | No change |
| … data donation for medical research in general (Q12)? | 67 (22.6%) | 7 (2.4%) | 220 (74.3%) | 6 (7.5%) | 5 (6.3%) | 69 (86.3%) |
| … the use of personal data for medical research by commercial institutions (Q13)? | 44 (14.9%) | 17 (5.7%) | 233 (78.7%) | 4 (5.0%) | 8 (10.0%) | 68 (85.0%) |

^a^ Two patients in the ‘pro data donation’ subgroup did not answer questions 12 and 13.

Supplementary Table 2: Results of Survey 2

| **Survey 2 Q3:** **There are currently considerations in Germany to legally allow medical research on pseudonymised data without the prior consent of patients, unless the patient objects to such use. The objection should be as simple as possible, e.g. possible to assert when visiting a doctor or a pharmacy. Would you agree to such a regulation?** | Fully agree | Rather agree | Rather disagree | Fully disagree |
| --- | --- | --- | --- | --- |
|  | 28 (21.2%) | 71 (53.8%) | 22 (16.7%) | 11 (8.3%) |

| **Survey 2 Q4: Do you agree that every citizen (diseased, or not) has a duty to contribute to medical research?** | Fully agree | Rather agree | Rather disagree | Fully disagree |
| --- | --- | --- | --- | --- |
| Pro data donation^a^ (n=99) | 25 (25.3%) | 65 (65.7%) | 8 (8.0%) | 1 (1.0%) |
| Contra data donation^a^ (n=33) | 2 (6.1%) | 11 (33.3%) | 15 (45.5%) | 5 (15.2%) |

^a^ For the purpose of the analysis of the survey 2 results, the 99 patients who answered either “Do fully agree” or “Do rather agree” to Q3 were categorized as ‘pro data donation’ whereas the 33 patients who answered “Do rather disagree” or “Do fully disagree” were categorized as ‘contra data donation’.

| **Survey 2 Q5: Where should medical data that were donated for medical research be stored (multiple answers possible)?^a^** | Site of original data acquisition only (e.g. clinic, GP) | Nation-wide centralized database | Respective research institution | Nowhere (owing to rejection of data donation) | Unknown |
| --- | --- | --- | --- | --- | --- |
|  | 37 (37.4%) | 75 (75.8%) | 23 (23.2%) | 1 (1.0%) | 2 (2.0%) |

^a^ Only the responses of the 99 patients in the ‘pro data donation’ subgroup are shown.

| **Survey 2 Q6: Who should be allowed use of data donated for medical research (multiple answer possible)?^a^** | Universities and public research institutions | | Commercial research institutions (pharma companies, insurance companies) | | Nobody (owing to rejection of data donation) | Unknown |
| --- | --- | --- | --- | --- | --- | --- |
|  | Germany, EU | Outside EU | Germany, EU | Outside EU |  |  |
|  | 91 (91.9%) | 24 (24.1%) | 14 (14.1%) | 7 (7.6%) | 1 (1.0%) | 6 (6.1%) |

^a^ Only the responses of the 99 patients in the ‘pro data donation’ subgroup are shown.

Questionnaire Survey 1

(Hospital Logo, University Logo)

Liebe Patientin, lieber Patient,

wir möchten gerne mehr über **Ihre Ansicht zur sogenannten „Datenspende**“ erfahren, die eine Nutzung von Patientendaten für die medizinische Forschung ermöglicht, ohne hierfür jedes Mal die Erlaubnis der Betroffenen einzuholen. Zu diesem Zweck haben wir den vorliegenden Fragebogen entwickelt und möchten Sie bitten, diesen spontan und ohne langes Nachdenken auszufüllen. Die Antworten werden von uns gesammelt und anschließend für eine wissenschaftliche Studie genutzt.

Die Beantwortung des Fragebogens wird nur ungefähr **8 Minuten** in Anspruch nehmen. **Ihre Teilnahme ist freiwillig**. Sie können die Teilnahme ohne Angabe von Gründen verweigern, ohne dass Ihnen daraus Nachteile entstehen.

Der Datenschutz wird bei der Umfrage gewahrt**. Die Auswertung des Fragebogens erfolgt anonym. Daher bitten wir Sie, den Fragebogen nicht mit Ihrem Namen zu versehen oder zu unterschreiben.**

Sollten Sie Fragen haben, wenden Sie sich bitte an unsere Kollegin vor Ort oder kontaktieren uns telefonisch unter **XXXXXX** oder per E-Mail an **XXXXXXXXX**

**Wenn Sie teilnehmen, geben Sie den ausgefüllten Fragebogen bitte anschließend an der Rezeption ab.**

Vielen Dank für Ihre Aufmerksamkeit.

**Geschäftsbereich Medizinethik - Institut für Experimentelle Medizin**

**Christian-Albrechts-Universität zu Kiel - Arnold-Heller-Str. 3, Haus 28 - 24105 Kiel**

─────────────────────────────────────────────────────────────────────────────

*Wir bitten Sie um Verständnis, dass im Folgenden aus Gründen der besseren Lesbarkeit zumeist die männliche Form verwendet wird.*

**Worum geht es in diesem Fragebogen geht?**

Medizinische Daten, die für die Forschung interessant und hilfreich sein könnten, fallen an vielen Stellen an - nicht nur in Krankenhäusern und Arztpraxen, sondern auch bei Krankenkassen, Apotheken und Versicherungen. Und immer häufiger produzieren Menschen gesundheitsrelevante Daten auch selbst, z.B. durch medizinische Apps auf ihrem Mobiltelefon.

Zurzeit arbeiten Experten daran, diese großen Datenmengen für die Forschung nutzbar zu machen. Technisch scheint dies kein großes Problem zu sein. Aus organisatorischen Gründen ist es jedoch schwierig (und meistens sogar unmöglich), von jedem Patienten eine Einwilligung in die Nutzung der bereits vorliegenden Daten einzuholen. Daher wird momentan über die Einführung einer **Datenspende** diskutiert, bei der Ihre persönlichen **Gesundheitsdaten unentgeltlich und nach eingehender Prüfung des Verwendungszwecks für die medizinische Forschung** zur Verfügung gestellt werden, es sei denn, Sie widersprechen dem ausdrücklich.

**1. Bitte nennen Sie Ihr Alter und Geschlecht.**

Alter (in Jahren): _________

Geschlecht:  männlich  weiblich  divers

**2. Bitte nennen Sie uns Ihren höchsten, bislang erreichten Bildungsabschluss.**

Haupt- bzw. Volksschule  Mittlere Reife  Abitur oder Fachabitur

kein Schulabschluss  anderer Abschluss: ___________________

**3. Welche der folgenden Aussagen zur medizinischen Forschung treffen Ihrer Ansicht nach derzeit zu?**

3.1. Daten aus der klinischen Routineversorgung können sinnvoll für die medizinische Forschung genutzt werden.

ja  nein  weiß ich nicht

3.2. Patientendaten dürfen ohne Einwilligung der Patienten nur von deren behandelnden Ärzten für die Forschung genutzt werden.

ja  nein  weiß ich nicht

3.3. Daten von deutschen Patienten dürfen auch für Forschung im Ausland genutzt werden.

ja  nein  weiß ich nicht

3.4. Universitäten arbeiten in der medizinischen Forschung auch mit kommerziellen Unternehmen zusammen.

ja  nein  weiß ich nicht

3.5. Aus wissenschaftlichen Veröffentlichungen können leicht Rückschlüsse auf meine Person gezogen werden.

ja  nein  weiß ich nicht

3.6. Wenn medizinische Daten in der Forschung genutzt werden, können sie nicht mehr zum Patienten zurückverfolgt werden.

ja  nein  weiß ich nicht

**4. Wie ist Ihre persönliche Einstellung zu medizinischer Forschung?**

4.1. Wer als Patient von medizinischer Forschung profitiert, sollte selbst einen Beitrag zur Forschung leisten.

| stimme gar nicht zu | stimme eher nicht zu | stimme eher zu | stimme voll zu |
| --- | --- | --- | --- |
|  |  |  |  |

4.2. Jeder Bürger (egal, ob krank oder nicht) hat die Pflicht, zur Verbesserung der medizinischen Forschung beizutragen.

| stimme gar nicht zu | stimme eher nicht zu | stimme eher zu | stimme voll zu |
| --- | --- | --- | --- |
|  |  |  |  |

4.3. Medizinische Forschung sollte mit persönlichen Vorteilen für die beteiligten Patienten verbunden sein.

| stimme gar nicht zu | stimme eher nicht zu | stimme eher zu | stimme voll zu |
| --- | --- | --- | --- |
|  |  |  |  |

4.4. In der Öffentlichkeit sollten mehr Informationen zu einzelnen Forschungsprojekten verfügbar sein.

| stimme gar nicht zu | stimme eher nicht zu | stimme eher zu | stimme voll zu |
| --- | --- | --- | --- |
|  |  |  |  |

4.5. Die Entscheidung über die Nutzung von Patientendaten für die medizinische Forschung darf nicht allein der Wissenschaft überlassen werden. Daran sollten auch Außenstehende (z.B. Selbsthilfegruppen, Kirchen oder Verbände) beteiligt sein.

| stimme gar nicht zu | stimme eher nicht zu | stimme eher zu | stimme voll zu |
| --- | --- | --- | --- |
|  |  |  |  |

4.6. Patientendaten sollten auch für medizinische Forschung in kommerziellen Unternehmen verfügbar sein.

| stimme gar nicht zu | stimme eher nicht zu | stimme eher zu | stimme voll zu |
| --- | --- | --- | --- |
|  |  |  |  |

**5. Es ist denkbar, dass persönliche Gesundheitsdaten wie z.B. Ihre Krankheitsgeschichte, Untersuchungsergebnisse oder Röntgenbilder zukünftig in einer digitalen Gesundheitsakte gespeichert werden.** **Wären Sie damit einverstanden, dass diese Daten in Form einer „Datenspende“ unentgeltlich und unter Wahrung des Datenschutzes für medizinische Forschungszwecke zur Verfügung gestellt werden, ohne dass Sie hierfür jedes Mal erneut um Erlaubnis gefragt werden?**

| voll einverstanden | eher einverstanden | eher nicht einverstanden | nicht einverstanden |
| --- | --- | --- | --- |
|  |  |  |  |

**6. Wären Sie damit einverstanden, dass auch von Ihnen selbst erhobene Daten (z.B. aus mobilen medizinischen Geräten oder Mobiltelefonen) der Forschung als Datenspende unentgeltlich und unter Wahrung des Datenschutzes zur Verfügung gestellt werden, ohne dass Sie hierfür jedes Mal erneut um Erlaubnis gefragt werden?**

| voll einverstanden | eher einverstanden | eher nicht einverstanden | nicht einverstanden |
| --- | --- | --- | --- |
|  |  |  |  |

**7. Wo sollten die medizinischen Daten, die Sie für Forschungszwecke spenden, gespeichert werden dürfen (Mehrfachnennung möglich)?**

nur an der Stelle, wo die Daten ursprünglich erhoben wurden (z.B. Klinik, Hausarzt)

in einer bundesweiten zentralen Forschungsdatenbank
 bei der jeweiligen Forschungsstelle
 nirgendwo, weil ich die Datenspende ablehne
 weiß ich nicht

**8. Wer sollte mit Ihren gespendeten medizinischen Daten forschen dürfen (Mehrfachnennung möglich)?**

Universitäten und öffentliche Forschungseinrichtungen in Deutschland

Universitäten und öffentliche Forschungseinrichtungen im Ausland

kommerzielle Unternehmen (z.B. Versicherungen, Pharmafirmen) in Deutschland

kommerzielle Unternehmen (z.B. Versicherungen, Pharmafirmen) im Ausland

niemand, weil ich die Datenspende ablehne

weiß ich nicht

*Die folgenden Fragen beziehen sich auf Ihre Einstellung zur Nutzung Ihrer gespendeten Daten für die Forschung in kommerziellen Unternehmen.*

*Hätten Sie Vorbehalte gegen eine solche Nutzung?*

*Wenn Sie Vorbehalte haben, oder wenn Sie die Datenspende grundsätzlich ablehnen, bitte Fragen 9 - 13 beantworten; wenn Sie keine Vorbehalte haben, bitte Fragen 10 -13 beantworten.*

**9. Welche Vorbehalte haben Sie gegenüber der Nutzung Ihrer Daten für die Forschung in kommerziellen Unternehmen (Mehrfachnennung möglich)?** Ich denke nicht, dass meine Daten bei solchen Unternehmen ausreichend geschützt sind.
 Ich möchte nicht, dass solche Unternehmen mit meinen Daten Profit erzielen.
 Ich denke nicht, dass solche Unternehmen für das Gemeinwohl forschen.
 Ich denke nicht, dass kommerzielle medizinische Forschung hinreichend wichtig ist.
 Ich lehne die Datenspende grundsätzlich ab.

**10. Wie könnte man Vorbehalten gegenüber der Nutzung von Patientendaten zur Forschung in kommerziellen Unternehmen begegnen (Mehrfachnennung möglich)?**

Es muss sichergestellt werden, dass die Daten nicht verkauft oder weiterverkauft werden.

Die Bedingungen für die Nutzung der Daten müssen durch ein Gesetz geregelt sein.

Die Datennutzung muss durch eine unabhängige Einrichtung kontrolliert werden.

Die Datennutzung der Daten muss durch eine staatliche Einrichtung kontrolliert werden.

Informationen über die Datennutzung müssen öffentlich verfügbar sein (z.B. in einem
 Register).

Der Datenschutz in den Unternehmen muss regelmäßig unabhängig kontrolliert werden.

Die Daten dürfen nicht in den Unternehmen selbst gespeichert werden.

**11. Bislang werden Patienten um ihre Einwilligung in die Datennutzung für die Forschung gebeten, wenn Sie zur Behandlung in eine Klinik kommen. Finden Sie das gut, oder würden Sie diese Entscheidung lieber bei anderer Gelegenheit treffen?**

Ich möchte diese Entscheidung auch weiterhin als Patient in der Klink treffen.

Ich möchte diese Entscheidung als Patient, aber außerhalb der Klinik, treffen.

Ich möchte diese Entscheidung treffen, bevor ich krank werde.

**12. Wie hat sich Ihre Einstellung zur Datenspende durch die Corona-Epidemie verändert?**

positiv  negativ  keine Änderung

**13. Wie hat sich Ihre Einstellung zur Nutzung ihrer medizinischen Daten für die Forschung in kommerziellen Unternehmen durch die Corona-Epidemie verändert?**

positiv  negativ  keine Änderung

**Vielen Dank für Ihre Teilnahme.**

Questionnaire Survey 2

(Hospital Logo, University Logo)

Liebe Patientin, lieber Patient,

wir würden gerne mehr über **Ihre Meinung zur sogenannten „Datenspende**“ erfahren. Deshalb bitten wir Sie, den vorliegenden Fragebogen spontan und ohne langes Nachdenken auszufüllen. Die Antworten werden von uns gesammelt und anschließend in einer wissenschaftlichen Studie ausgewertet.

Die Beantwortung des Fragebogens dauert ungefähr 5 Minuten. **Ihre Teilnahme ist freiwillig**. Sie kann von Ihnen ohne Angabe von Gründen verweigert werden, ohne dass Ihnen daraus Nachteile entstehen.

Um den Datenschutz zu wahren, erfolgt die Umfrage anonym. **Daher bitten wir Sie, den Fragebogen nicht mit Ihrem Namen zu versehen oder zu unterschreiben.**

Sollten Sie noch Fragen haben, wenden Sie sich bitte an unsere Kollegin vor Ort oder kontaktieren uns telefonisch unter **XXXX** oder per E-Mail **XXXX**.

**Wenn Sie teilnehmen möchten, geben Sie den ausgefüllten Fragebogen bitte anschließend an der Rezeption ab.**

Vielen Dank für Ihre Aufmerksamkeit.

**Geschäftsbereich Medizinethik - Institut für Experimentelle Medizin**

**Christian-Albrechts-Universität zu Kiel - Arnold-Heller-Str. 3, Haus 28 - 24105 Kiel**

─────────────────────────────────────────────────────────────────────────────

*Wir bitten Sie um Verständnis, dass im Folgenden aus Gründen der besseren Lesbarkeit zumeist die männliche Form verwendet wird.*

**Worum geht es in diesem Fragebogen?**

Daten, die für die medizinische Forschung wertvoll und interessant sein könnten, fallen an vielen Stellen im täglichen Leben an - nicht nur in Krankenhäusern und Arztpraxen, sondern auch bei Krankenkassen, Apotheken und Versicherungen.

Zurzeit arbeiten Experten daran, diese **Daten für die medizinische Forschung nutzbar zu machen**. Aus organisatorischen Gründen ist es jedoch schwierig (und häufig sogar unmöglich), jeden Patienten jedes Mal um seine Einwilligung in die Nutzung seiner Daten für die Forschung zu bitten.

Daher wird die Einführung einer sogenannten „**Datenspende“** diskutiert, bei der persönliche **Gesundheitsdaten der medizinischen Forschung automatisch zur Verfügung stehen**, es sei denn, der Patient widerspricht dem ausdrücklich. Die Nutzung der Daten soll nur **pseudonymisiert, unentgeltlich und nur nach eingehender, unabhängiger Prüfung des Zwecks der Nutzung** erfolgen.

**1. Bitte nennen Sie Ihr Alter und Geschlecht.**

Alter (in Jahren): _________

Geschlecht:  männlich  weiblich  divers

**2. Bitte nennen Sie uns Ihren höchsten, bislang erreichten Bildungsabschluss.**

Haupt- bzw. Volksschule  Mittlere Reife  Abitur oder Fachabitur

kein Schulabschluss  anderer Abschluss: ___________________

**3.** **Derzeit gibt es in Deutschland Überlegungen, die medizinische Forschung an pseudonymisierten Daten ohne vorherige Einwilligung der Patienten gesetzlich zu erlauben, es sei denn, der Patient widerspricht dieser Nutzung. Dieser Widerspruch soll möglichst einfach sein, z.B. bei einem Besuch beim Arzt oder in einer Apotheke.
Wie fänden Sie eine solche gesetzliche Regelung?**

| völlig falsch | eher falsch | eher richtig | völlig richtig |
| --- | --- | --- | --- |
|  |  |  |  |

**4. Wie beurteilen Sie die Aussage, dass jeder Bürger (egal, ob krank oder nicht) die Pflicht hat, zur Verbesserung der medizinischen Forschung beizutragen?**

**Ich finde diese Aussage**

| völlig falsch | eher falsch | eher richtig | völlig richtig |
| --- | --- | --- | --- |
|  |  |  |  |

**5. Wo sollten medizinische Daten, die für Forschungszwecke gespendet wurden, Ihrer Meinung nach gespeichert werden dürfen (Mehrfachnennung möglich)?**

nur an der Stelle, wo die Daten ursprünglich erhoben wurden (z.B. Klinik, Hausarzt)

in einer bundesweiten zentralen Forschungsdatenbank

bei der jeweiligen forschenden Stelle

nirgendwo, weil ich die Datenspende ablehne

weiß ich nicht

**6. Wer sollte mit medizinischen Daten, die für Forschungszwecke gespendet wurden, forschen dürfen (Mehrfachnennung möglich)?**

Universitäten und öffentliche Forschungseinrichtungen in Deutschland und der EU

Universitäten und öffentliche Forschungseinrichtungen außerhalb der EU

kommerzielle Unternehmen (z.B. Versicherungen, Pharmafirmen) in Deutschland und der EU

kommerzielle Unternehmen (z.B. Versicherungen, Pharmafirmen) außerhalb der EU

niemand, weil ich die Datenspende ablehne

weiß ich nicht

**Vielen Dank für Ihre Teilnahme.**
